# Medical and Philosophical News

**Published:** 1781-06

**Authors:** 


We are informed that M. Andry, an ingenious physician at Paris, has lately given the inspissated juice of the *saponaria officinalis*, or *sopewort*, to a great number of patients labouring under gonorrhœa. The patients took about half an ounce of this medicine daily, and in general, a cure was effected in about a fortnight without the assistance of any other remedy.

